# Cytochrome P450_BM-3_ and P450 11A1 retain Compound I (FeO^3+^) chemistry with electrophilic substrates poised for Compound 0 (Fe^3+^O_2_^−^) reactions

**DOI:** 10.1016/j.jbc.2025.110378

**Published:** 2025-06-14

**Authors:** Kevin D. McCarty, Yasuhiro Tateishi, F. Peter Guengerich

**Affiliations:** Department of Biochemistry, Vanderbilt University School of Medicine, Nashville, Tennessee, USA

**Keywords:** cytochrome P450, CYP, BM-3, 11A1, fatty acid oxidation, steroid oxidation, enzyme mechanism, Compound I, Compound 0

## Abstract

The catalytic cycle of cytochrome P450 (P450) enzymes involves ferric peroxide anion (Fe^3+^O_2_^−^, Compound 0) and perferryl oxygen (FeO^3+^, Compound I) intermediates. Compound I is generally viewed as responsible for most P450-catalyzed oxidations, but Compound 0 has been implicated in the oxidation of some carbonyl compounds, particularly deformylation reactions. We considered the hypothesis that Compound 0 could also attack other electrophilic carbon atoms and accordingly positioned keto groups at preferred hydroxylation sites of substrates for two P450s with well-defined catalytic reactions, bacterial P450_BM-3_ (102A1), and human P450 11A1. The predicted products of Compound I and Compound 0 reactions were analyzed. With the normally preferred **ω**-1 site blocked, P450_BM-3_ oxidized 12-oxotridecanoic acid (12-oxo C13:0) only at the **ω**-2 position (yielding 11-hydroxy,12-oxotridecanoic acid), indicative of a Compound I oxidation. P450 11A1 is highly selective for catalyzing the 22*R*-hydroxylation of cholesterol (and some other sterols) in the first step of its overall side-chain cleavage reaction. With 22-oxocholesterol as the substrate, P450 11A1 (slowly) generated only 23-hydroxy,22-oxocholesterol, indicative of Compound I oxidation. Neither P450 generated the products expected from nucleophilic Compound 0 reactions. We conclude that the strategic placement of electrophilic oxo substituents at sites of substrate hydroxylation failed to divert the oxidation mechanism to a Compound 0 pathway with either enzyme. Instead, the Compound I mechanism—blocked at the preferred reaction site—was redirected to neighboring carbons, suggesting that the basis for Compound 0-mediated reactions lies in chemical properties of the enzyme rather than those of the substrate.

Cytochrome P450 (P450) enzymes are the major catalysts involved in biological oxidations (1). These enzymes are distributed throughout all kingdoms of life and are the major catalysts involved in the oxidations of pharmaceuticals, steroids, chemical carcinogens, fat-soluble vitamins, pesticides, industrial chemicals, pollutants, and natural products (1, 2, 3, 4). The repertoire includes not only simple carbon hydroxylations but also a diverse set of more complex oxidations, *e.g.* ring expansion, desaturation, ester cleavage, Diels-Alder conjugation, C-C, C-N, C-S, and C-O bond coupling (3, 5, 6, 7, 8, 9). Further, modifications of these enzymes by directed evolution/molecular breeding have further expanded the breadth of oxidations catalyzed by simple P450s (such as P450_BM-3_) to include reactions as exotic as C-Si bond cleavage and carbene insertion (10, 11, 12, 13).

An expanded view of the basic P450 catalytic cycle (Fig. 1) includes two reactive entities, the first of which is termed Compound 0 (formally Fe^3+^O_2_^−^ in its relevant unprotonated form) and can be converted by protonation and loss of H_2_O to yield the second oxidant Compound I, the species considered to be involved in most P450 oxidations (5, 14).

**Figure 1:**
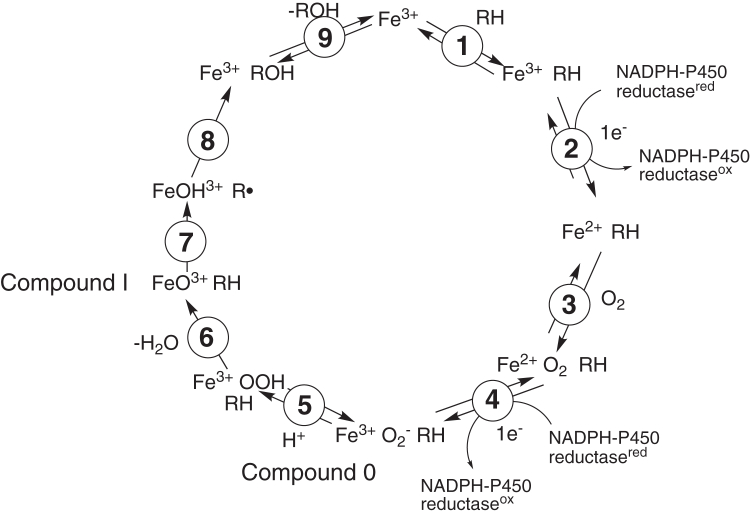
**Classical mechanism for P450-catalyzedoxidations.**

However, a role for Compound 0 has been proposed to explain some P450 oxidations (5, 15). The distinction between these reactive species in the catalysis of some oxidations has been difficult to establish and rather controversial in some cases (5, 16). Our own recent work with P450s 2B4 and 19A1 has established a rather exclusive role for Compound 0 in some oxidations (17, 18), but we have presented evidence that *both* Compound 0 and Compound I reactions can occur in the oxidative 14α-deformylation of dihydrolanosterol catalyzed by P450 Family 51 enzymes (19). In the P450 51 work, we concluded that a modified version of the classic catalytic cycle (Fig. 1) was in order (Fig. 2).

**Figure 2:**
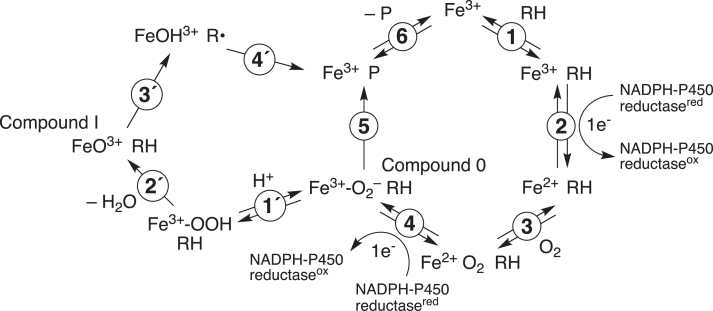
**Generalized mechanism for P450-catalyzedoxidations.**

In this modified mechanism (Fig. 2), formation of Compound 0 in the presence of an electrophilic substrate (which has an electron deficient carbonyl group) enables (in theory) two competing reactions whereby the Compound 0 nucleophile either attacks the substrate (leading directly to product formation, Step 5) or is protonated to form Compound I (Steps 1′, 2′), which may subsequently generate the same reaction product (or others) *via* a different mechanism (Steps 3′, 4′). These studies raise the question of whether the positioning of an electrophilic carbonyl group at the site of a P450 hydroxylation might be a sufficient change to redirect the P450 oxidation mechanism from Compound I to Compound 0 chemistry (Fig. 2).

We selected two well-characterized P450s, bacterial P450_BM-3_ (102A1) and human P450 11A1. Although the physiological role of P450_BM-3_ in *Bacillus megaterium* has yet to be established, P450_BM-3_ and its mutants are of considerable interest in biocatalysis applications (11, 20), including synthesis of fine chemicals (21), drug metabolites (22, 23), and potentially commodity chemicals (11, 24). P450 11A1, the cholesterol side chain cleavage enzyme that catalyzes the initial step in mammalian steroid biosynthesis (Fig. 3), is of interest not only in endocrinology but also industrial applications, notably the bulk synthesis of steroids. Cleavage of the side chain of inexpensive plant sterols such as β-sitosterol (25) is catalyzed by this enzyme (26, 27), facilitating the synthesis of valuable steroids, and *B*. *megaterium* systems that express P450 11A1 have been used for this purpose (28).

**Figure 3:**
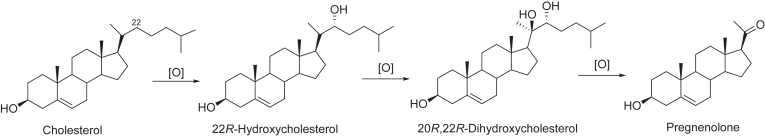
**Three-step oxidation of cholesterol topregnenolone by P450 11A1.**

We synthesized the cognate substrates of these two P450 enzymes with a ketone group positioned at the normal carbon site for hydroxylation, a generally accepted Compound I reaction. Different products were expected for Compound 0 reactions at each site (Figs. 4 and 5), and these were synthesized. Our results indicated no diversion from Compound I to Compound 0 chemistry with either of those two P450s, in that the enzymes only yielded alternative Compound I products.

**Figure 4:**
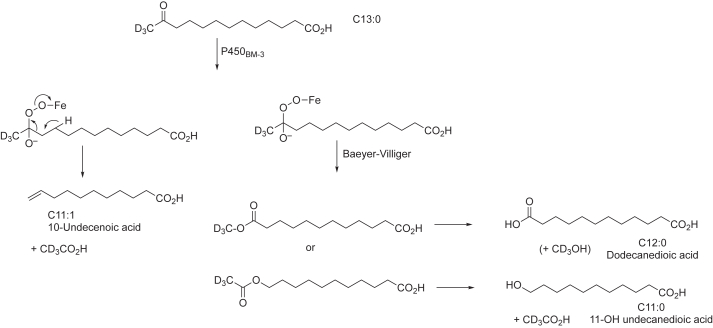
**Potential P450**_**BM-3**_**Compound 0 reactions with12-oxotridecanoicacid.**

**Figure 5:**
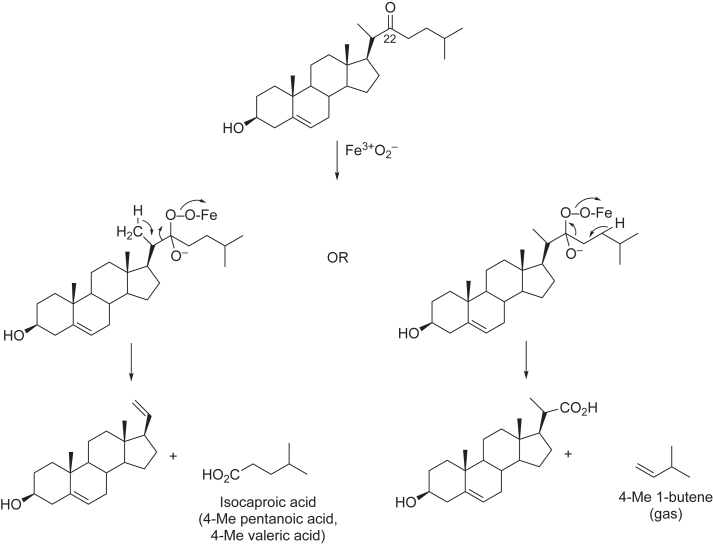
**Potential P450 11A1 Compound 0 reactions with22-oxocholesterol.**

## Results

### P450_BM-3_ strategy

**Figure 6:**
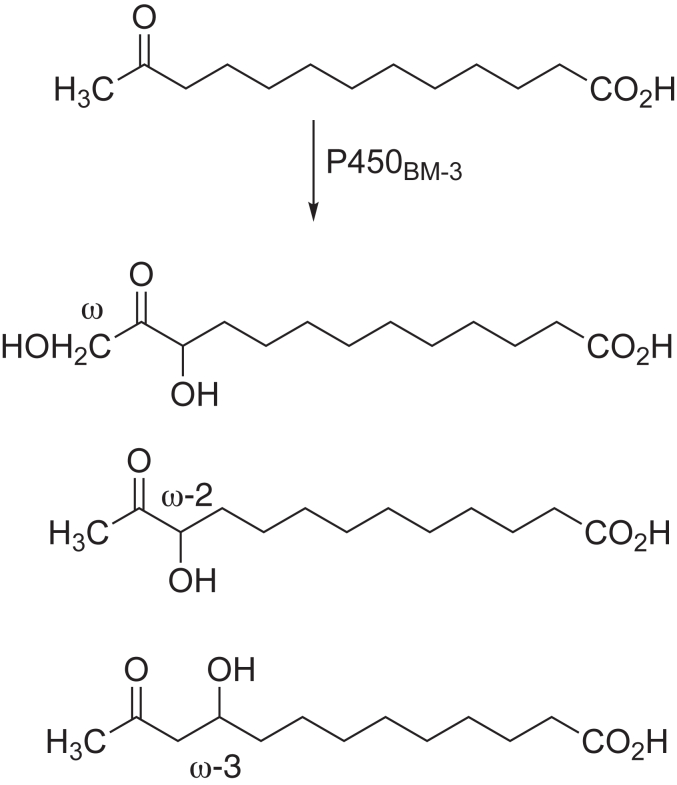
**Potential P450**_**BM-3**_**Compound I reactions with 12-oxotridecanoic acid.**

P450_BM-3_ (CYP102A1) is an extensively studied bacterial P450 that can catalyze the hydroxylation of some medium-to long-chain fatty acids and alkyl sulfates at high rates (29). It catalyzes hydroxylation of dodecanoic (lauric) acid (C12:0) and tetradecanoic (myristic) acid at the ω-1, ω-2, and ω-3 carbons but not at the ω position, yielding hydroxy products (*i.e.*, 11-, 10-, and 9-hydroxy dodecanoic acid, Figure 6), in a molar ratio of ∼ 3:1:1 (29, 30). A C-terminal CD_3_ group was included to facilitate MS analysis of any deuterated acetic acid that might be formed in a Compound 0 (C-C cleavage) reaction, with the additional advantage that it would retard any potential Compound I hydroxylation at the ω-position. Potential Compound 0 products of 12-oxotridecanoic acid that might be expected are shown in Figure 4. ω-Hydroxylation is disfavored, due to the inherently more difficult hydrogen abstraction associated with the C-H bond dissociation energy of a methyl group relative to a methylene (98 vs 94 kcal mol^-1^) (31), and to our knowledge, ω-hydroxylation by P450_BM-3_ has never been reported (11). Some other P450s can catalyze fatty acid ω-hydroxylation due to the restrictive shapes of their active sites, with a tight channel positioning the (ω) CH_3_ group near the FeO complex (32).

### Synthesis of substrates

12-Oxotridecanoic acid (12-oxo C13:0) was selected as the test substrate in light of the known rates of hydroxylation of lauric acid (C12:0) and myristic acid (C14:0) (29), solubility, and availability of starting materials for synthesis. The substitution of deuterium at the ω position (Fig. 6) could further retard any ω-hydroxylation and provide a method of analyzing acetic acid if that were a suspected product.

The six-step synthesis of the substrate [13-^2^H_3_]-12-oxotridecanoic acid (Supporting Information) was relatively straightforward, utilizing a Wittig reagent and a Markovnikov hydration. The final product was not dissolved in basic solutions with water or alcoholic solvents to avoid loss of deuterium due to enol formation.

### P450_BM-3_ product analysis

P450-catalyzed reactions rely on electrons (from NADPH) delivered to the enzyme by flavoproteins. In general, the flavoprotein exists as a distinct enzyme (redox partner, *e.g.* NADPH-P450 reductase, adrenodoxin reductase, *etc.*); however, the bacterial P450_BM-3_ structure consists of a flavin domain fused to a P450 domain—a property that renders the enzyme self-sufficient.

**Figure 7:**
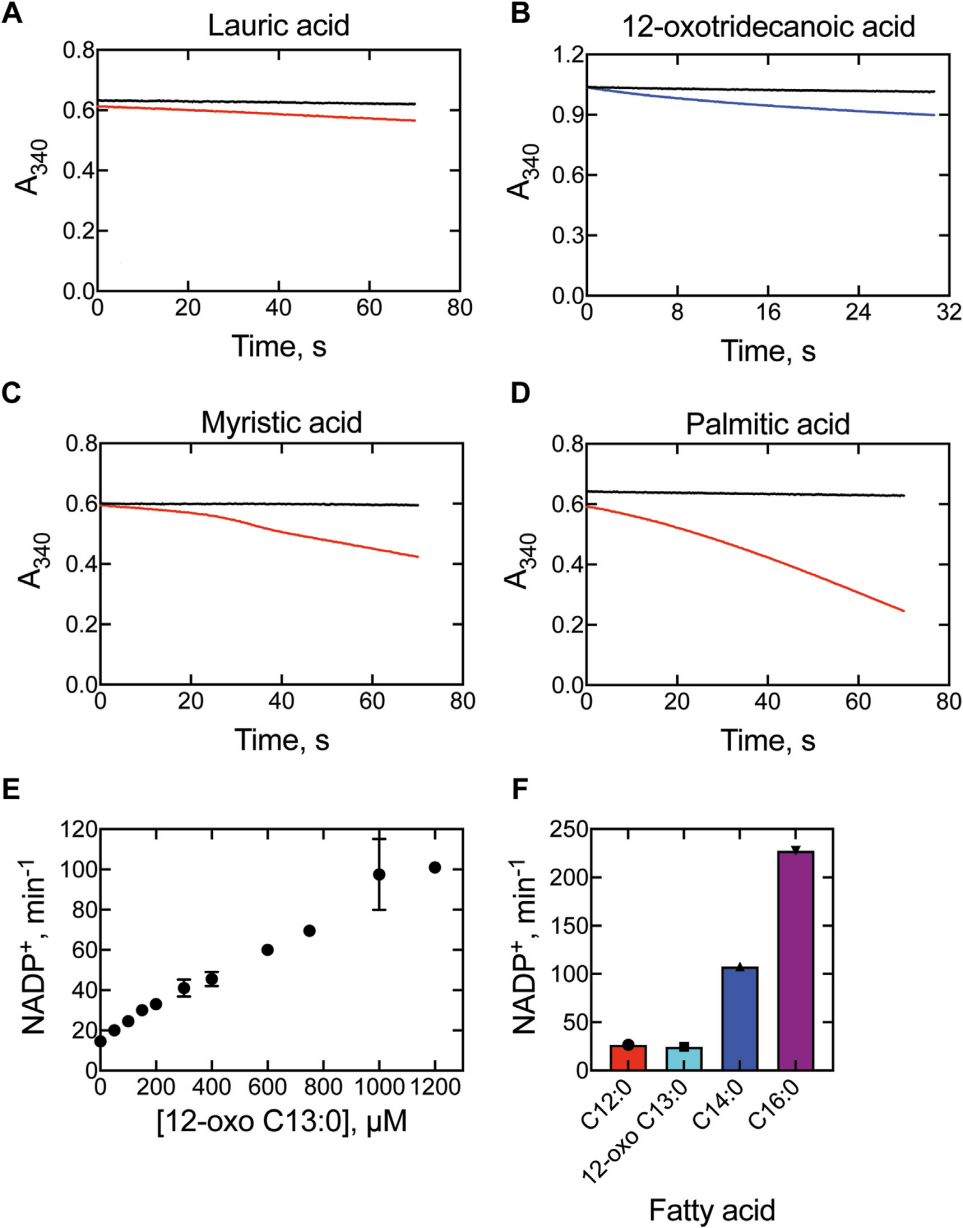
**Stimulation of NADPH oxidation by P450_BM-3_ and fatty acids**. P450_BM-3_ (0.25 μM) and NADPH (150 μM) were incubated with: *A*, lauric acid (C12:0), *B*, 12-oxotridecanoic acid (12-oxo C13:0), *C*, myristic acid (C14:0), and *D*, palmitic acid (C16:0) at fatty acid concentrations of 100 μM (*red*) or 1000 μM (*blue*). *E*, plot of NADPH oxidation rate vs. concentration of 12-oxotridecanoic acid (0–1200 μM). *F*, comparison of NADPH oxidation rates (n = 1) stimulated by each fatty acid (100 μM). *Black lines* (Parts *A*–*D*): no fatty acid added.

When the binding of a substrate to a P450 enzyme triggers NADPH consumption (oxidation to NADP^+^) but fails to stimulate product formation (substrate oxidation) the reaction is considered uncoupled, in that product formation is slow compared to NADPH consumption. The ability of 12-oxo C13:0 to stimulate NADPH oxidation by P450_BM-3_ was assessed and compared to three known substrates of the enzyme: lauric acid (C12:0), myristic acid (C14:0), and palmitic acid (C16:0). The C12:0, 12-oxo C13:0, C14:0, and C16:0 fatty acids stimulated NADPH oxidation, at rates of 27 min^-1^, 25 min^-1^, 108 min^-1^, and 228 min^-1^, respectively, when measured with 100 μM substrate (Fig. 7, *A*–*D*). The concentration dependence of this effect was further probed with 12-oxo C13:0, which was not saturable and reached a maximum rate of 101 nmol NADPH oxidized min^-1^ (nmol P450_BM-3_)^-1^ (Fig. 7*E*). Thus, 12-oxo C13:0 was able to enter the P450_BM-3_ active site and behave like a substrate of the enzyme, stimulating NADPH oxidation at a reasonable rate (relative to the natural substrates, Fig. 7*F*).

**Figure 8:**
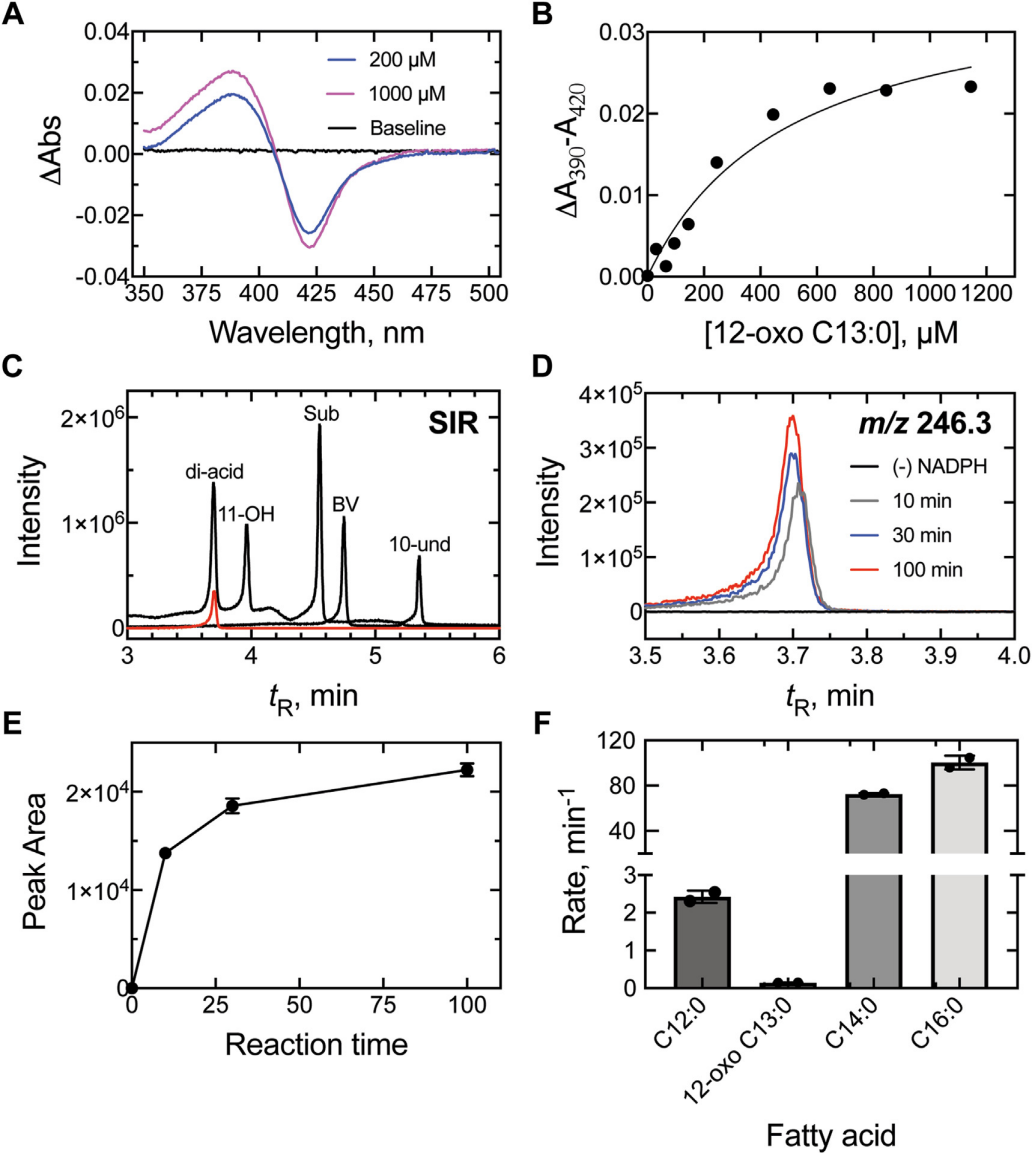
**Fatty acid binding and oxidation kinetics of P450_BM-3_**. *A,* binding spectra of P450_BM-3_ (1 μM) and 12-oxotridecanoic acid (200 μM (*blue*) and 1000 μM (*magenta*) (bound vs free P450 difference spectra). *B,* binding isotherm of 12-oxotridecanoic acid (12-oxo C13:0, 0–1200 μM) and P450_BM-3_ (1 μM). Substrate binding is plotted as Δ*A*_390_-*A*_420_. *C,* selected ion (SIR) chromatograms of proposed P450 Compound 0 products (*black*) and the [M-H+16]^−^ product of the P450_BM-3_ reaction with 12-oxo C13:0. Di-acid, dodecanedioic acid; 11-OH, 11-hydroxy undecanoic acid; Sub, substrate (12-oxotridecanoic acid); BV, Baeyer-Villiger product; 10-und, 10-undecanoic acid (Fig. 4). *D,* chromatogram of P450_BM-3_ incubations (10 min (*gray*), 30 min (*blue*), and 90 min (*red*)) with 12-oxo C13:0. The [M-H+16]^−^ product ion (*m/z* 246.3) channel is shown. *E,* peak area integration (n = 2) of the *m/z* 246.3 product ion. *F,* fatty acid hydroxylation rate of P450_BM-3_ with lauric (C12:0), 12-oxotridecanoic (12-oxo C13:0), myristic (C14:0), and palmitic (C16:0) acids (n = 2).

To assess the extent to which NADPH consumption was coupled to product formation (fatty acid oxidation), 12-oxo C13:0 was tested as a substrate of P450_BM-3_. Binding of 12-oxo C13:0 to P450_BM-3_-induced a classical Type I P450 spectral change (*i.e.* from 418 nm (low spin) to 390 nm (high-spin)) that was observed to be saturable with a *K*_d_ of ∼250 μM (Fig. 8, *A* and *B*). When P450_BM-3_ was incubated with [13-*d*_3_]-12-oxotridecanoic acid (200 μM, near *K*_d_), LC-MS analysis revealed only one NADPH- and time-dependent product, which did not migrate (on reversed-phase HPLC) with any of the proposed Compound 0 C-C bond cleavage products (Figs. 4, 8). The product was characterized with *m/z* 246.3 (parent ion, +16 amu higher than the substrate) indicating a single oxidation, and the possibility of a Compound 0 Baeyer-Villiger oxygen atom insertion (Fig. 4) (19, 33) was excluded (by *t*_R_) following synthesis and analysis of the putative Baeyer-Villiger product (Fig. 8*C*).

The oxidation rate of 12-oxo C13:0 was calculated and compared to the hydroxylation rates of the C12:0 to C16:0 fatty acids (Fig. 8*F*). P450_BM-3_ oxidized 12-oxo C13:0 at a rate of ∼0.15 min^-1^, which was between 16-fold and 670-fold slower than the rates observed for C12:0, C14:0, and C16:0 fatty acids (2.4 min^-1^, 73 min^-1^, and 100 min^-1^, respectively). When rates of product formation (Fig. 8) and NADPH consumption (Fig. 7) were compared, it was revealed that only ∼0.6% of the electrons (from NADPH) were used for 12-oxo C13:0 oxygenation, with the rest presumably yielding H_2_O_2_ or H_2_O. This figure compares to values of ∼9%, ∼68%, and ∼44% calculated for C12:0, C14:0, and C16:0 fatty acids (based on standard curves of 12-OH C12:0 or 16-OH C16:0), respectively, indicating that the 12-oxo C13:0 reaction was highly inefficient (uncoupled). Thus, in Figure 2, Compound 0 is formed but unable to do Step 5 and only proceeds through Steps 1′-4′ slowly.

**Figure 9:**
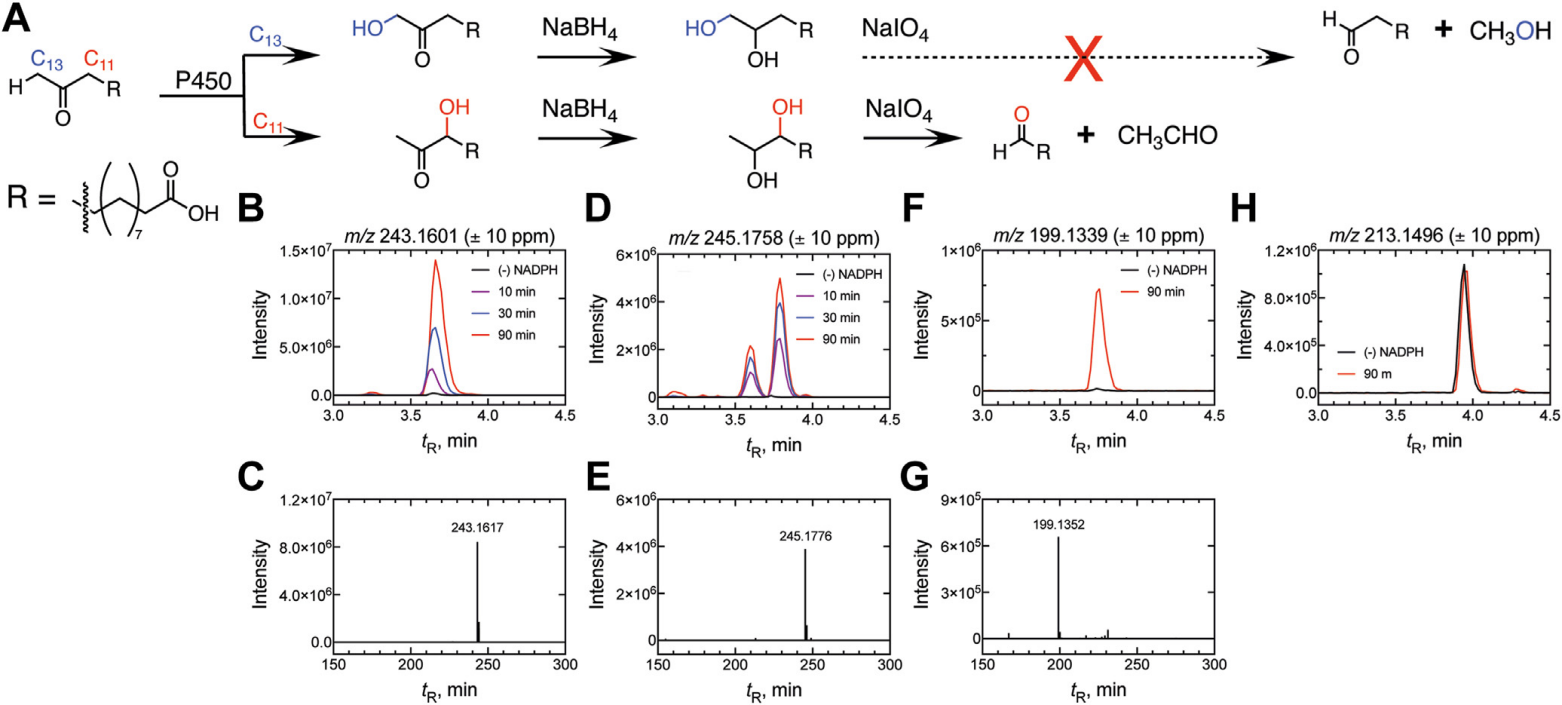
**Characterization of P450_BM-3_ fatty acid hydroxylation product**. *A*, scheme depicting the formation of both possible hydroxylation products (at carbons 11 (C_11_, *red*) and 13 (C_13_, *blue*) (R = C_10_H_18_O_2_^-^). The products of the P450_BM-3_ reaction were reduced (with NaBH_4_) and the product (a diol) was cleaved (with HIO_4_) to yield the possible products displayed. A *dashed arrow* and *red cross* indicate a molecule that was not detected. *B*, *D*, *F*, and *H*, chromatograms of a P450_BM-3_ incubation with 12-oxotridecanoic acid with (*red*) and without (*black*) initiation by NADPH (*A, m/z* channel 243.1601) that was subsequently treated with *D*, NaBH_4_ (*m/z* channel 245.1758) and then *F* and *H,* HIO_4_ (*m/z* channels of 199.1339 and *m/z* 213.1496, respectively). *C*, *E*, and *G, m/z* spectra collected at the retention times (*t*_R_) of the products identified in *B*, *D*, and *F*, respectively. All chromatograms are extracted with a 10 ppm *m/z* window from the theoretical *m/z* of the product. The corresponding chemical structures of each product are displayed.

The single oxidation product of the P450_BM-3_ reaction with 12-oxo C13:0 was suspected to be a Compound I-mediated hydroxylation (Compound 0 products were not detected (rate < 2 × 10^-5^ min^-1^), Figure 8, *C* and *D*). Given that P450_BM-3_ catalyzes reactions at the fatty acid ω-1, ω-2, and ω-3 carbons, two products ((ω-2)-OH and (ω-3)-OH at carbons 11 (C-11), and 10 (C-10) respectively) of the reaction were feasible (with the ω-1 carbon blocked by a carbonyl) (Fig. 9*A*). We also included the possibility of ω-hydroxylation (at carbon-13 (C-13)), an unnatural P450_BM-3_ reaction, potentially resulting from diversion of the preferred reaction from the blocked ω-1 carbon. To account for a possible ω-hydroxylation product, a protiated (C-13 CH_3_) substrate was used (as opposed to the deuterated C-13 CD_3_) to avoid a potential isotope effect.

To identify the oxidized carbon, the product of the P450_BM-3_ reaction (Fig. 9, *B* and *C*) was reduced with NaBH_4_ (Fig. 9, *D* and *E*) and the resulting diol was cleaved with HIO_4_ (Fig. 9, *F* and *H*). This approach yields a 12-carbon aldehyde if the initial hydroxylation (by P450_BM-3_) was catalyzed at C-13 and an eleven-carbon aldehyde if at C-11 (Fig. 9*A*). Additionally, if initial hydroxylation was catalyzed at C-10 the NaBH_4_ reduction product (10,12-dihydroxytridecanoic acid) will not be cleaved with HIO_4_ treatment (as it is not a vicinal glycol). LC-MS analysis (negative ion electrospray) clearly identified one NADPH-dependent product of *m/z* 199.1352 (molecular ion [M-H]^-^) corresponding to the 11-carbon aldehyde. The 12-carbon aldehyde peak ([M-H]^-^ *m/z* 213.1496) was not NADPH-dependent (Fig. 9, *F* and *H*) (and is presumed to be a minor impurity resulting from the intermediacy of its ethyl ester in the synthetic scheme (Supporting Information, Scheme S1). Both diol peaks disappeared with HIO_4_ treatment, indicating that both were vicinal diols and excluding the possibility of a C-10 hydroxylation product.

We conclude that the carbonyl moiety in 12-oxo C13:0 did not divert the P450_BM-3_ oxidation mechanism to Compound 0 chemistry (Baeyer-Villiger chemistry or C-C bond cleavage). Instead, the carbonyl blocked the normal Compound I (ω-1)-hydroxylation reaction (the major reaction catalyzed with C12:0-C16:0 fatty acids) and drove (ω-2)-hydroxylation (but not ω-hydroxylation), albeit at a much slower rate.

### P450 11A1 strategy

We selected (human) P450 11A1, an enzyme that catalyzes the 3-step side-chain cleavage of cholesterol to form pregnenolone (Fig. 3). There is independent evidence for the involvement of Compound I in all three steps (34, 35, 36). The first step is the stereoselective (*R*) hydroxylation at C22. Accordingly, we considered 22-oxocholesterol as a substrate, in that the products generated might be diagnostic of Compound 0 chemistry (Fig. 5) depending on which β-proton (to the oxygen-bonded carbon) would be abstracted. The available literature had shown that P450 11A1 can tolerate some diversity of substrates (37, 38, 39, 40), including both the *R*- and *S*-isomers of 22-hydroxy cholesterol (41), and also that 22-oxocholesterol could bind to the enzyme (26, 42, 43).

### Syntheses for P450 11A1 reactions

The substrate 22-oxocholesterol was synthesized (previously commercially available (42, 43) but not currently), installing a carbonyl moiety at the carbon normally hydroxylated in the first step of cholesterol side chain cleavage. Although several syntheses of 22-oxocholesterol have been published (44, 45, 46, 47, 48), the historical first approach of these (Supporting Information Scheme S2, from the Fernholtz acid, 23,24-bisnor-5-cholenic acid-3β-ol) (44, 45) proved to be the most successful.

### P450 11A1 product analysis

22-Oxocholesterol had been reported to bind tightly to bovine P450 11A1 and behave as an inhibitor, with no products formed (43). The *K*_d_ was not reported in molarity, but comparison with our own measured *K*_d_ for cholesterol (0.12 μM) (49) would suggest ∼ 30 nM. Another group had reported that 22-oxocholesterol was actually a better substrate than cholesterol with bovine P450 11A1 (for conversion to pregnenolone) (42). Still another laboratory reported no formation of pregnenolone and a competitive *K*_i_ value of 29 μM for 22-oxocholesterol (26).

**Figure 10:**
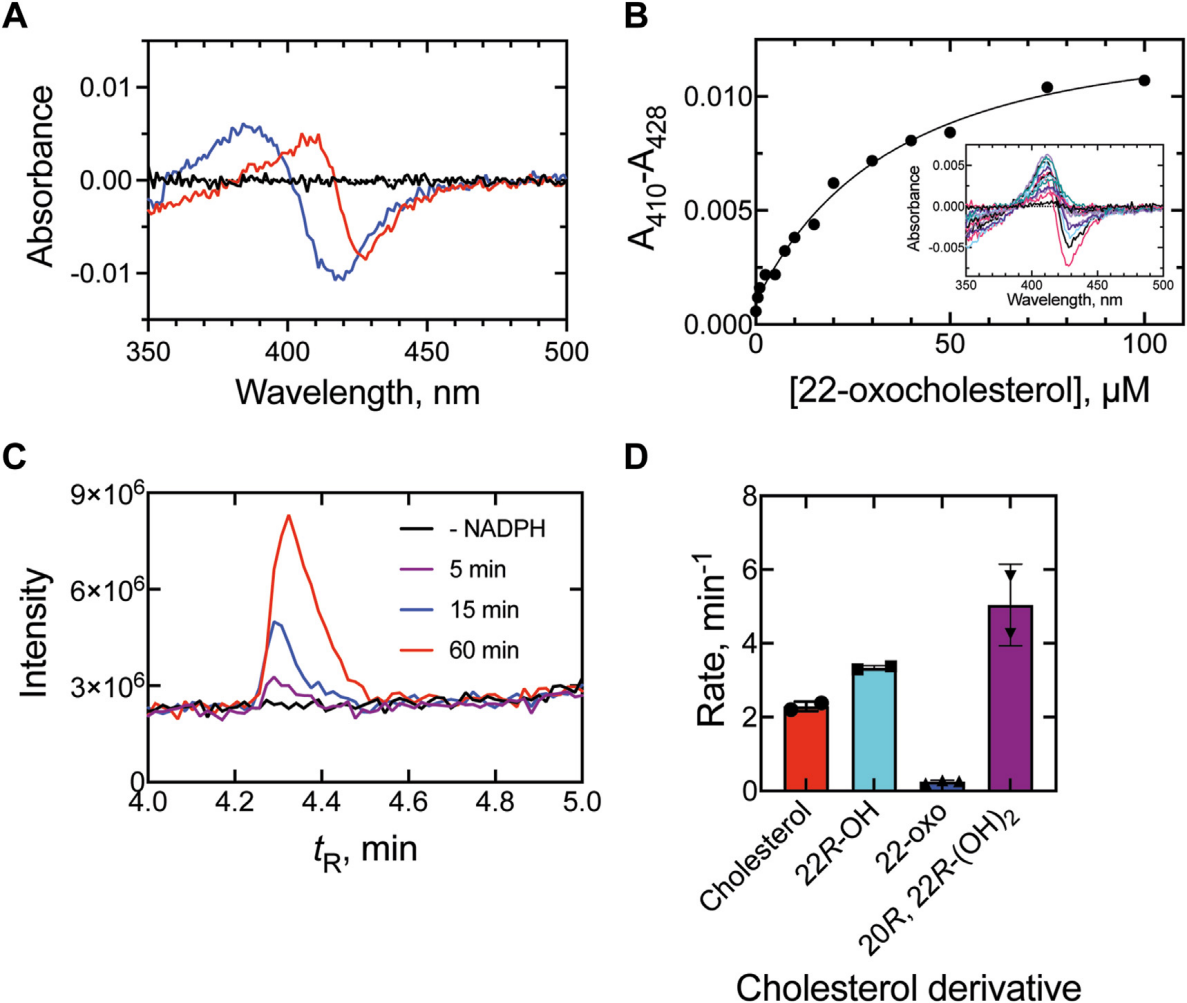
**Sterol binding and oxidation kinetics of P450 11A1**. *A,* binding spectra of P450 11A1 (1 μM) and 22-oxocholesterol (5 μM, *red*) that was then treated with cholesterol (5 μM, *blue*). *B,* binding isotherm of 22-oxocholesterol (0–100 μM) and P450 11A1 (1 μM). Substrate binding is plotted as Δ*A*_410_-*A*_428_. *C,* chromatogram of the oxidation product ([M+H-H_2_O]^+^ ion, *m/z* 399.3258 ± 5 ppm) of the P450 11A1 reaction (5, 15, and 60 min) with 22-oxocholesterol. *D,* comparison of 22-oxocholesterol hydroxylation rate (estimated from substrate consumption of three independent 60 min reactions with 25 μM substrate), to that of cholesterol, 22*R*-hydroxy cholesterol (22*R*-OH), and 20*R*,22*R*-dihydroxy cholesterol (from two replicates of 5 min reactions of 20 μM substrate).

22-Oxocholesterol bound to P450 11A1 with a rather unusual (potentially bathochromic) spectral change (Fig. 10*A*), reminiscent of some others in the literature (50). The spectral shift was not fully saturated at the tested concentrations (0–100 μM) and a *K*_d_ value of 43 ± 20 μM was estimated (Fig. 10*B*), >2000-fold weaker than the binding of 22*R*-OH cholesterol to the enzyme (49). As previously mentioned, a much tighter *K*_d_ for 22-oxocholesterol was reported (43); however, this value was determined with the bovine enzyme and was estimated from reverse Type I spectral changes, while we observed Type II spectral behavior with the human enzyme.

LC-MS analysis of incubations of P450 11A1 with 22-oxocholesterol revealed a +16 amu product that was both NADPH- and time-dependent (Fig. 10*C*) but was formed at a slow rate (0.25 ± 0.04 min^-1^), <4% of the *k*_cat_ measured for cholesterol 22-hydroxylation (Fig. 10*D*), assessed by substrate depletion. Neither of the proposed Compound 0 steroid products (Fig. 5) was detected, indicating a maximal rate of formation <0.003 min^-1^ (based on limits of detection).

Neither Compound 0 nor Compound I reactions were observed with the use of oxygen surrogates, including the single-atom oxygen donor (Compound I mimic) iodosylbenzene and the potential double-atom donors (Compound 0 mimics) H_2_O_2_ and cumene hydroperoxide.

**Figure 11:**
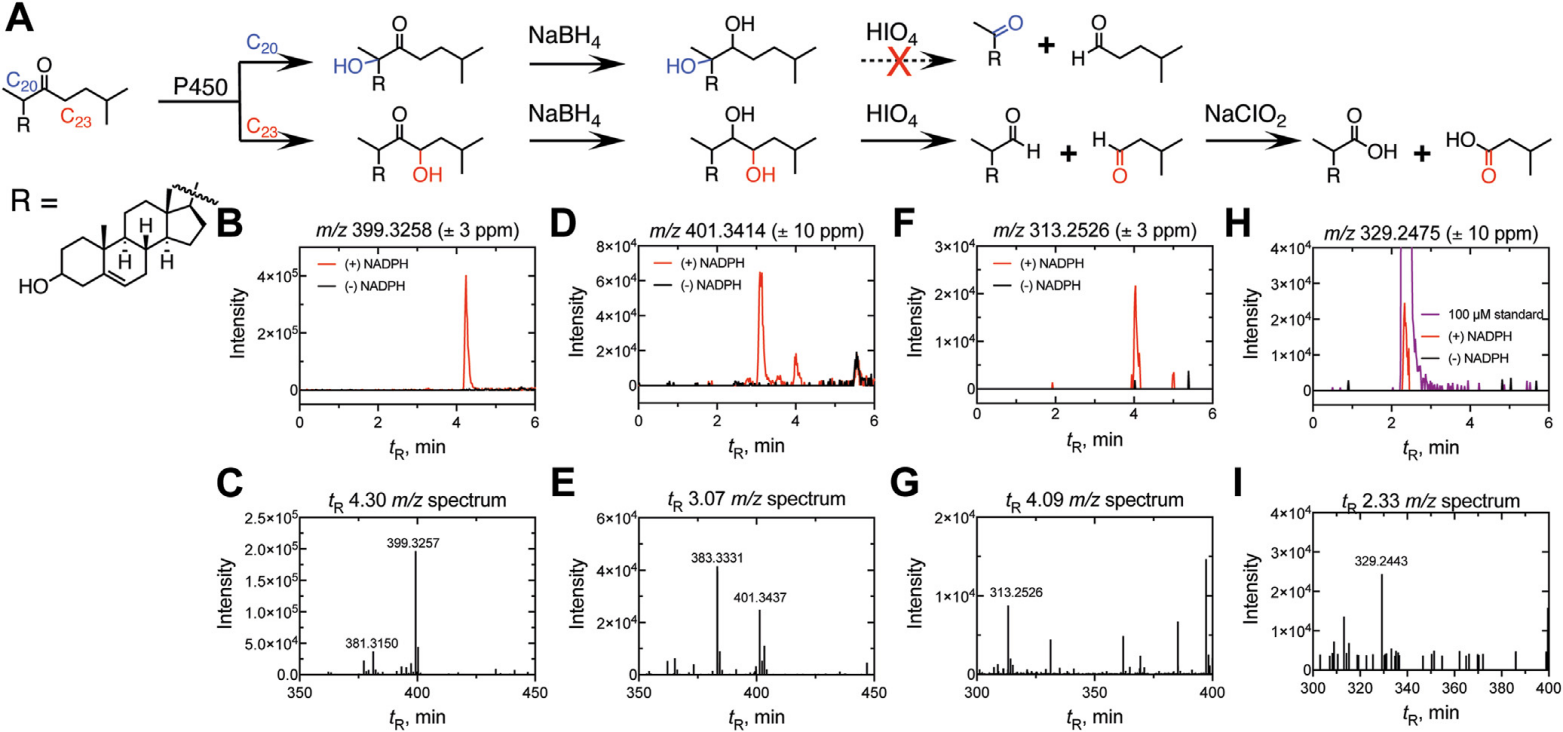
**Characterization of P450 11A1 sterol oxidation product.***A*, scheme depicting the formation of both possible hydroxylation products (at carbons 23 (C_23_, *red*) and 20 (C_20_, *blue*) (R = C_19_H_28_^+^). The products of the P450 11A1 hydroxylation reaction were subsequently reduced (with NBH_4_) and the product (a diol) was cleaved (with HIO_4_) to yield the possible products displayed. The resulting aldehyde was oxidized (with NaClO_2_) to an acid, the identity of which was confirmed with an authentic commercial standard. A *dashed arrow* and *red cross* indicate a molecule that was not detected. *B, D*, *F*, and *H*, APCI-MS chromatograms (of the [M+H-H_2_O]^+^ ions) of a P450 11A1 incubation with 22-oxocholesterol with (*red*) and without (*black*) initiation by NADPH (*B, m/z* channel 399.3258 ± 3 ppm) that was subsequently treated with NaBH_4_ (*D*, *m/z* channel 401.3414 ± 10 ppm), cleaved with HIO_4_ (*F*, *m/z* channel 313.2526) and oxidized with NaClO_2_ (*H*, *m/z* channel 329.2475) (33, 51). *C*, *E*, *G,* and *I*, *m/z* spectra collected at the retention time (*t*_R_) of the product identified in Parts *B*, *D*, *F*, and *H*, respectively.

The product of the P450 11A1 reaction was suspected to involve hydroxylation of 22-oxocholesterol, and two carbons (carbons 20 (C-20) and 23 (C-23)) were both potentially feasible sites for the reaction. To characterize the site of P450 11A1 oxidation, the same approach (Fig. 11*A*) was employed as in the work with P450_BM-3_ above. The product of the P450 11A1 reaction (Fig. 11, *B* and *C*) was reduced with NaBH_4_ to a diol (Fig. 11, *D* and *E*) which was cleaved by HIO_4_ to yield 23,24-bisnor-5-cholenic aldehyde-3β-ol (Fig. 11, *F* and *G*). The identity of each intermediate was confirmed by positive-ion APCI-HRMS, with the cleavage product (an aldehyde) observed at *m/z* 313.2526 (Δ 0.0 ppm for C_22_H_33_O^+^, the [M+H-H_2_O]^+^ ion) (Fig. 11). The aldehyde was subsequently oxidized to an acid with NaClO_2_ to yield an acid (33, 51), the identity of which was confirmed by LC-HRMS (Δ 9.8 ppm for *m/z* C_22_H_33_O_2_^+^, the [M+H-H_2_O]^+^ base peak, Fig. 11, *G* and *H*) and by verification of LC-HRMS retention time (and mass spectrum) with an authentic commercial standard of 23,24-bisnor-5-cholenic acid-3β-ol (Fig. 11*H*, used in the synthesis of 22-ketocholesterol).

Although C-20 hydroxylation (of 22*R*-OH cholesterol) is a natural reaction in the side chain cleavage of cholesterol, the installation of the carbonyl moiety at C-22 instead steered the Compound I reaction to the opposite neighboring carbon (C-23). We conclude that the 22-oxo group did not activate Compound 0 chemistry (Fig. 5) and instead blocked the normal Compound I-mediated hydroxylation at C-22, attenuating the reaction rate by ∼96% (compared to normal cholesterol C-22 hydroxylation).

## Discussion

There has been considerable interest in understanding the catalytic mechanisms of the oxidations that P450 enzymes catalyze and in rationalizing the formation of unusual reaction products (5, 7, 11, 16). Although the early catalysis literature in this field involved a variety of potential intermediate oxidation species (*e.g.*, superoxide anion (52), hydroxy radical (53)), eventually the dogma came to center on a perferryl oxygen complex (formally FeO^3+^), referred to as Compound I, and the general concept of the hydrogen abstraction/oxygen rebound (Fig. 1) (14, 54). P450 oxidations include not only simple hydroxylation but also numerous rearrangements, heteroatom oxygenations, C-C bond scissions, C-C, C-N, C-S, and C-O bond formations, and other reactions (3, 7, 8, 16). Many of these can be rationalized in the context of perferryl oxygen reactions, that is, involving Compound I (FeO^3+^) (3, 6, 7, 8, 14, 15, 16, 30, 54). However, some P450-catalyzed oxidations can be explained by mechanisms involving Compound 0, the ferric peroxide anion Fe^3+^O_2_^–^ (Fig. 2) (15). This is a nucleophilic species, in contrast to the electrophilic Compound I (FeO^3+^). A number of approaches have been utilized to discern between Compound I and Compound 0 mechanisms, the advantages and limitations of which we have analyzed at length in the course of our own ^18^O_2_ labeling studies (17, 18, 19) and summarized in two recent review articles (55, 56). Analysis of reaction products is an appropriate approach when Compound 0 chemistry may generate different products than Compound I chemistry. In the latter case, the enzyme is operating in its normal NADPH-dependent setting, either with a typical or unusual substrate, which is the approach used here.

We positioned carbonyl (ketone) groups at well-characterized hydroxylation positions of classic substrates of bacterial P450_BM-3_ and human P450 11A1 (Figs. 4 and 5), severely attenuating catalysis in both cases (Figs. 8*F*, 10*D*). The hypothesis was whether the ketone substituents would divert the oxidation reaction of both substrates from a Compound I-mediated hydroxylation to Compound 0 chemistry (Fig. 2), with (proposed) potential products derived from oxygen atom insertion (Baeyer-Villiger chemistry) and C-C bond scission. Those reactions did not occur (at a detectable rate) with P450_BM-3_ or P450 11A1. Both enzymes, which are reported to have somewhat relaxed substrate selectivity in some instances (11, 30, 40, 57, 58), instead retained Compound I chemistry but directed the site of oxidation to the neighboring carbon, catalyzing the reactions at much slower rates than at the normally preferred carbon (Figs. 9 and 11).

The question can be raised as to whether a ketone shows enough electronic separation (δ^+^C=Oδ^–^) to invite an attack by Compound 0 intermediate (Fe^3+^O_2_^−^) on the carbon atom. The attack of Compound 0 on an electrophilic site is proposed to be competitive with the rate of protonation of Compound 0 to generate Compound I (Fig. 2). It is possible that the electronic charge separation in a ketone may not be as great as in aldehydes, for which there is the most evidence for Compound 0 reactions (17, 18, 19). A Compound 0 mechanism, with an attack on a ketone, has been advanced for the P450 17A1 side-chain cleavage of 17α-hydroxy progesterone and 17α-hydroxy pregnenolone (59, 60, 61, 62, 63), although alternate (Compound I) mechanisms have also been proposed (64, 65) and ^18^O-labeling experiments are not unambiguous in discerning the mechanisms with α-hydroxy ketones (α-ketols) (16, 65, 66, 67, 68). If aldehydes are really favored in Compound 0-mediated oxidations (relative to ketones), how are aldehydes oxidized to carboxylic acids? These are fairly common reactions and both Compound I and Compound 0 reactions can be proposed, but it may be possible to discern these in future studies.

Although P450 11A1 has been shown to be an efficient, processive enzyme with high catalytic specificity in cholesterol side-chain cleavage (49), its use of some alternate substrates is known. For instance, several vitamin D oxidation reactions are known (37, 38, 39) and both rat and human P450 11A1 were implicated in the conversion of a structurally-unrelated drug candidate to a toxic product (40). Thus, the potential for involvement of P450 11A1 in other reactions certainly exists.

22-Oxocholesterol has been considered before, in a biological context. 22*S*-Hydroxy cholesterol was oxidized to 22-oxocholesterol by bovine adrenal mitochondria in an NADPH-dependent reaction that was not inhibited by carbon monoxide and therefore presumed not to involve P450 (69). The significance of this reaction is unclear, in that P450 11A1 only oxidizes cholesterol to the *R*-isomer of 22-hydroxy cholesterol (although the synthetic *S*-isomer is also a substrate for subsequent carbon-20 hydroxylation), and the oxidation on to pregnenolone (Fig. 3) is highly processive (49). 22-Oxocholesterol was reported to be a strong inhibitor of bovine P450 11A1 and not to be converted to pregnenolone or to any other products, although the method of analysis was focused only on hydrazone derivatives (43). These results were in contrast to a report that bovine P450 11A1 converted 22-oxocholesterol to pregnenolone (42), actually faster than from cholesterol (2.2 min^-1^). Still another group reported no formation of pregnenolone (and a competitive *K*_i_ value of 29 μM for 22-oxocholesterol) (26). Kautsky *et al.* (45) administered [23-^14^C]-labeled 22-oxocholesterol to guinea pigs and found labeled isovaleric acid in the livers but not labeled isocaproic acid (Fig. 5) in the adrenals, leading the authors to conclude that C22-C23 bond cleavage had occurred and that 22-oxocholesterol was not an intermediate in the normal synthesis of pregnenolone. This final product in an experimental animal model is consistent with the 23-hydroxylation reaction we observed with recombinant human P450 11A1, although the course of cleavage is not known and the biological relevance is unknown.

Previous work with bacterial P450_cam_ (P450 101A1) attempted to address a similar hypothesis (through modifying the protein, not the substrate), altering a conserved threonine residue (Thr-252) to block the Compound I formation step (70, 71). This mutation did attenuate the camphor hydroxylation reaction by 78% to 95%. Mutating the conserved Thr-268 of P450_BM-3_ decreased total dodecanoic acid hydroxylation by 85% (product expressed as sum of (ω-1)-OH, (ω-2)-OH, and (ω-3)-OH products) (72). However, that substitution also decreased the rate of O_2_ consumption by 75%, and subsequent work by others led to the conclusion that the role of Thr-268 is one of substrate positioning, not assistance of protonation (73, 74, 75). It is conceivable that some of the >1500 P450_BM-3_ mutants that have been made (11) might do Compound 0 chemistry, but predicting which do it *a priori* is not obvious, and we have not tried an experimental approach to using libraries of these. Although many natural variants and site-directed mutants of P450 11A1 have been considered (76, 77), none have been identified that disrupt the proton delivery system (the substitution T291S had little effect on the catalytic activity (76)). Whether Compound 0 chemistry can be activated by mutagenesis of either enzyme would thus require identification of residues solely involved in Compound I formation and not substrate binding or juxtaposition. The crystal structures of P450_BM-3_ are not very revealing in that the site(s) of oxygenation are 7 to 8 Å away from the iron and not useful in predicting productive orientations of catalysis (78, 79, 80). X-ray crystal structures of P450 11A1 have been obtained with cholesterol, the reaction intermediates 22*R*-hydroxy and 20*R*,22*R*-dihydroxy cholesterol, and the alternate substrate 20*S*-hydroxy cholesterol (81, 82), which all appear to be well accommodated with the basic structure. Strushkevich *et al.* (82) concluded that “…the C22 position is more dynamic than C20…”, and thus 22-oxocholesterol should be a reasonable probe of the ability of the enzyme to alter its chemistry. There are deficiencies in utilizing crystal structures to predict P450 mechanisms. For instance, our X-ray crystal structures of human P450 51A1 with lanosterol (14α-methyl) and the 14α-formyl lanosterol intermediate are superimposable but the initial hydroxylation of lanosterol is a simple Compound I reaction while the oxidative deformylation of the aldehyde is dominated by Compound 0 chemistry (19).

There are presumably more P450 reactions that can be rationalized in the context of Compound 0 chemistry, although the fraction is probably low compared to Compound I. For instance, a minor pathway of the metabolism of the drug brepocitinib was published recently, with speculation that a C-C cleavage involved the addition of a singlet oxygen (^1^Δ_O2_) to an olefin, formation of a strained dioxetane, and loss of H_2_O_2_ (83). Some of these proposed steps are unusual in biochemistry, particularly the generation and use of singlet oxygen in the liver. A more satisfying mechanism can be written with the use of Compound 0, with the attack on an electrophilic carbon (α to the carbonyl on the pyrazoline ring) to generate the observed products.

We conclude that both P450_BM-3_ and P450 11A1 continued to drive Compound I chemistry when presented with a “pro-Compound 0” (electrophilic) substrate derivative (Fig. 2). This is presumably due to Step 1´ (protonation) outcompeting Step 5 in Figure 2, even when Step 1′ was attenuated. Moreover, the efficiency of the oxygenation process (compared to the natural substrates) was already severely impaired, in that the substrate (of the P450_BM-3_ reaction) stimulated abortive NADPH oxidation (at rates comparable to C12:0) but formed considerably less product (Figs. 7 and 8). Thus, the Compound I mechanisms of both P450_BM-3_ and P450 11A1 were maintained even in the presence of electrophilic substrates and disruption of the protonation step (Step 1′ in Fig. 2) still did not poise the enzymes for Compound 0 chemistry.

Formation of Compound 0 constitutes a junction in the P450 catalytic cycle from which formation of Compound I competes with direct substrate attack (and product formation, Fig. 2). Our present findings suggest that both P450_BM-3_ and P450 11A1 are poised to favor a particular pathway based on enzymatic properties (structure, active site chemistry) rather than substrate properties, and that engineering molecules may not be a viable approach to hone and leverage Compound 0 chemistry, which may be desirable in some biocatalytic applications. While these results certainly raise the question as to how some P450s (2B4, 19A1, and 51A1) are able to include both mechanisms in their catalytic repertoire—including (in some cases) to catalyze the same reaction (*e.g.*, P450 51 enzymes)—we consider that the mechanistic competition is more complex than the simple proximity of electron-rich vs. electron-deficient substrate sites to Compound I or Compound 0 in the P450 active site.

## Experimental procedures

### Chemicals

See Supporting Information for syntheses of [13-^2^H_3_]-12-oxotridecanoic acid, 11-acetoxy undecanoic acid, 22-oxocholesterol, and pregna-5,20-dien-3β-ol and corresponding HRMS and ^1^H and ^13^C NMR spectra (Supporting Figs. S1-S5).

### Enzymes

P450_BM-3_ was expressed from a cDNA vector (originally obtained from the late Armand J. Fulco) and purified as described earlier (29). The electrophoretic and spectral properties have also been described previously (29), and a polyacrylamide gel of all studied proteins is presented in Supporting Figure S6. The expression, purification, and characterization of human P450 11A1 (36, 49, 84) and redox partners adrenodoxin (Adx) (85) and adrenodoxin reductase (AdR) (86) have also been described previously.

### NMR spectra

NMR spectra were acquired in CDCl_3_ using Bruker 400 and 600 MHz instruments in the Vanderbilt facility.

### Enzyme assays

Spectral binding measurements of P450 11A1 and P450_BM-3_ and NADPH oxidation assays with P450_BM-3_ were as previously described (29).

#### Assays with P450_BM-3_

Incubations for product analysis were done with 1 μM P450_BM-3_ in 50 mM potassium phosphate buffer (pH 7.4) and 200 μM substrate (added either from [13,13,13]-*d*_3_-12-oxotridecanoic acid dissolved in DMSO (to 10 mM stock) or 12-oxotridecanoic acid dissolved in 5 mM K_2_CO_3_ (to 2 mM stock)). Reactions (1.0 ml) were preincubated (5 min, 37 °C) before initiation with a NADPH-generating system (87) and were allowed to proceed (0–90 min, 37 °C) before quenching by the addition of CH_2_Cl_2_ (5 ml). Samples were centrifuged (1000*g*, 5 min) to separate layers, and the organic (lower) phase (4 ml) was transferred to fresh vials. Solvent was removed under a steady stream of nitrogen gas, the dried residue was reconstituted in CH_3_OH (0.10 ml), and the solution was transferred to autosampler glass vials for LC-MS analysis. Samples were analyzed initially by low resolution LC-MS and later by high resolution LC-MS as described.

Reactions of P450_BM-3_ with lauric, myristic, and palmitic acids were conducted as described above with the modifications that the reaction times were decreased (to 5 min, 1 min, and 1 min, respectively), the enzyme concentration was reduced to 0.05 μM, and that substrates were prepared in C_2_H_5_OH.

#### Assays with P450 11A1

Incubations were performed as previously described (49) with 0.25 μM P450 11A1, 0.5 μM AdR, and 10 μM Adx (molar component ratio of 1:2:40) in 50 mM potassium HEPES buffer (pH 7.4). 22-Oxocholesterol was initially prepared as a 10 mM stock in C_2_H_5_OH and diluted to 250 μM (1:40) in (2-hydroxypropyl)-β-cyclodextrin (45%, w/v) to yield the working stock solution, which was diluted (1:10, v/v) in the reaction mixture (to 25 μM). Reactions (0.5 ml) were preincubated (5 min, 37 °C) before initiation with a NADPH-generating system (87) and were allowed to proceed (0–60 min, 37 °C) before quenching by the addition of CH_2_Cl_2_ (5 ml). Samples were centrifuged (1000*g*, 5 min) to separate layers, and the organic (lower) phase (4 ml) was transferred to fresh vials. Solvent was removed under a steady stream of nitrogen gas, the dried residue was reconstituted in CH_3_OH (0.10 ml), and the solution was transferred to autosampler glass vials for high resolution LC-APCI-MS analysis as described below.

#### Assays with P450 11A1 and oxygen surrogates

Assays with P450 11A1 and the oxygen surrogates iodosylbenzene, cumene hydroperoxide (CuOOH), and H_2_O_2_ were conducted as previously described (18, 19). Briefly, solutions of P450 11A1 (0.25 μM) and 22-oxocholesterol (25 μM) were prepared in potassium HEPES buffer (50 mM, pH 7.4) and incubated with the Compound 0 mimics cumene hydroperoxide or H_2_O_2_ (each tested at both 0.1 mM and 1 mM concentrations) or the Compound I mimic iodosylbenzene (at 0.1 mM). Reactions (0.5 ml) were allowed to incubate (60 min, 37 °C) prior to quenching (note that the hyderoperoxides can also be involved in a Compound I mechanism). Samples were quenched, processed, and analyzed as described above.

### Characterization of P450 hydroxylation products

#### Reduction of keto groups with NaBH_4_

The products of the P450 reactions were extracted and solvent was removed under a stream of nitrogen gas (as described earlier). To the dried residue was added 5 mg of fresh NaBH_4_ powder and CH_3_OH (0.2 ml). The mixture was mixed with a vortex device and incubated (37 °C) for 30 min. H_2_O (0.25 ml) and CH_2_Cl_2_ (2 ml) were added (to the P450 11A1 reduction samples), and the products of the reaction were extracted into the solvent (CH_2_Cl_2_), the organic layer (1.6 ml) was removed, and the solvent was evaporated as described above. The extraction was repeated to maximize product recovery.

For the P450_BM-3_ reduction products, the final extraction step was omitted as it was observed that the product (a diol) was only poorly extracted into solvent. Instead, HCl (10 μl, 3 M) was added to the samples (to quench excess NaBH_4_) and the solution was brought to dryness under nitrogen gas.

#### Cleavage of vicinal diols with HIO_4_

The products of the NaBH_4_ reduction (a dried residue) were reconstituted in CH_3_OH (150 μl) and HIO_4_ (prepared as a 5 mg ml^-1^ solution in H_2_O) was added (150 μl) (49, 88). The solution was mixed with a vortex device and incubated (37 °C, 60 min) and transferred to autosampler vials. The products were analyzed by LC-MS (see “LC-MS analysis of products”).

#### Oxidation of aldehydes with NaClO_2_

The products of the P450 11A1 reaction (reduced with NaBH_4_ and cleaved with HIO_4_ reaction as described above) were oxidized to carboxylic acids (from aldehdyes) with NaClO_2_ *via* modification of existing protocols (33, 51, 89). Briefly, H_2_O (0.25 ml) was added to the HIO_4_ reaction solution, and the products were extracted into 2 volumes of CH_2_Cl_2_ (2 ml) as described above. The solvent was evaporated under a stream of nitrogen gas, and the dried residue was reconstituted in a solution of sulfanilamide (50 mM, added from a 100 mM solution in a 1:1 H_2_O:*tert*-butanol mixture, v/v), H_2_PO_4_ (25 mM), and NaClO_2_ (25 mM, added from a 400 mM solution in H_2_O). The reaction composition was strictly maintained at 50% aqueous *tert*-butanol to maintain solubility of all reaction components. The reaction was allowed to proceed overnight (∼20 h, 25 °C), after which H_2_O (0.25 ml) was added, and the products of the reaction were extracted into two volumes of CH_2_Cl_2_ (2 ml) as described above. The solvent was evaporated under a stream of nitrogen gas, and the dried residue was reconstituted in CH_3_OH (0.1 ml) for UPLC-APCI-HRMS as described below.

### LC-MS analysis of products

#### Low resolution LC-MS analysis

The products of the P450_BM-3_ reaction were injected (10 μl, held at 25 °C) using a Waters Acquity UPLC and separated (flow rate of 0.3 ml min^-1^) using a 2.1 mm × 50 mm (1.7 μm) Acquity BEH octadecylsilane (C_18_) column (held at 25 °C) with a gradient mobile phase of 10 mM NH_4_OAc dissolved in (A) 5% CH_3_CN in H_2_O and (B) CH_3_OH as follows (expressed as %B, v/v): 0 min, 25%; 0.5 min, 25%; 5.5 min, 100%; 8.5 min, 100%; 8.6 min, 25%; 10 min, 25%. The column eluate was introduced into a Waters QDa (single quadrupole) on-line mass spectrometer and ionized using negative-ion electrospray with a cone voltage of 30 V, a sampling frequency of 10 Hz, and scanning from *m/z* 100 to 500. Data were processed using the MassLynx software (Waters).

#### High resolution LC-MS analysis

##### Positive-ion APCI-HRMS of P450 11A1 products

The products of the P450 11A1 reaction were injected (10 μl, held at 25 °C) using a Waters Acquity UPLC and separated (flow rate 0.2 ml min^-1^) using a 2.1 mm × 100 mm (1.7 μm) Acquity BEH octadecylsilane (C_18_) column (held at 25 °C) with a gradient mobile phase of 0.1% HCO_2_H dissolved in (A) H_2_O and (B) CH_3_CN as follows (expressed as %B, v/v): 0 min, 70%; 0.5 min, 70%; 3 min, 100%; 8 min, 100%; 8.1 min, 70%; 10 min, 70%. The column eluate was subjected to APCI ionization (positive ion mode) in a Thermo Fisher Scientific LTQ XL Orbitrap mass spectrometer in the Vanderbilt Mass Spectrometry Research Core Facility, with a vaporizer temperature of 350 °C, a resolution setting of 60,000, and scanning from *m/z* 100 to 800. Data were processed using Xcalibur QualBrowser (Thermo Fisher Scientific) software (version 2.0.7).

##### Negative-ion ESI-HRMS of P450_BM-3_ products

P450_BM-3_ reaction products were injected and separated using the same LC conditions and mobile phase as described above (see “Low resolution LC-MS analysis”) and ionized with the same mass spectrometry conditions as described earlier (see “Positive-ion APCI-MS of P450 11A1 products”) with the modifications that ionization was performed using a heated electrospray source (negative-ion mode) with a vaporizer temperature of 300 °C and scanning from *m/z* 100 to 500.

## Data availability

Additional data are available in the Supporting Information and contain the syntheses of [13-^2^H_3_]-12-oxotridecanoic acid, 11-acetoxy undecanoic acid, 22-oxocholesterol, and pregna-5,20-dien-3β-ol, including NMR and selected mass spectra.

## Supporting information

This article contains supporting information. The references used are (44, 45, 46, 90, 91, 92, 93).

## Conflict of interest

The authors declare that they have no conflicts of interest with the contents of this article.
