# Supplementary material for: Cytochrome P450BM-3 and P450 11A1 retain Compound I (FeO3+) chemistry with electrophilic substrates poised for Compound 0 (Fe3+O2−) reactions
Source: J Biol Chem. 2025 Jun 14;301(7):110378. doi: 10.1016/j.jbc.2025.110378 (PMC12332404; doi:10.1016/j.jbc.2025.110378)
Supplement: Supporting Information [file mmc1.docx]

**Supporting Information**

**Cytochrome P450_BM-3_ and P450 11A1 retain Compound I (FeO^3+^) chemistry with electrophilic substrates poised for Compound 0 (Fe^3+^O_2_¯) reactions**

Kevin D. McCarty, Yasuhiro Tateishi, and F. Peter Guengerich*

Department of Biochemistry, Vanderbilt University School of Medicine, Nashville, Tennessee 37232-0146, United States

**Table of Contents**

**Synthetic procedures**

**Synthesis of [13-^2^H_3_]-12-oxotridecanoic acid**

**Scheme S1.** **Synthesis of [13-^2^H_3_]-12-oxotridecanoic acid.**  p. S-2

**Step 1.**  12-Hydroxydodecanoic acid ethyl ester. p. S-2

**Step 2.** 12-Oxododecanoic acid ethyl ester. p. S-2

**Step 3.** [13-^2^H_2_]-Tri-12-decenoic acid ethyl ester. p. S-3

**Step 4.** [13-^2^H_2_]-Tri-12-decenoic acid. p. S-3

**Step 5.** [13-^2^H_3_,12-^2^H]-12-Hydroxytridecanoic acid. p. S-3

**Step 6.** [13-^2^H_3_]-12-Oxotridecanoic acid. p. S-4

**Figure S1.** NMR spectra of 12-oxotridecanoic acid. p. S-5

**Synthesis of 11-acetoxy undecanoic acid** (Baeyer-Villiger intermediate) p. S-6

**Synthesis of 22-oxocholesterol**

**Scheme S2.** **Synthesis of 22-oxocholesterol.**  p. S-6

**Step 1.** 23,24-Bisnor-5-choleric acid-3β-ol (Fernholtz acid). p. S-6

**Step 2.**  p. S-7

**Step 3.**  p. S-7

**Step 4.**  p. S-7

**Figure S2.** HRMS of 22-oxocholesterol (APCI^+^). p. S-8

**Figure S3.** NMR spectra of 22-oxocholesterol. p. S-9

**Synthesis of pregna-5,20-dien-3β-ol**

**Scheme S3. Synthesis of pregna-5,20-dien-3β-ol** p. S-10

**Step 1.**  p. S-10

**Step 2.**  p. S-10

**Step 3.**  p. S-11

**Step 4.**  p. S-11

**Figure S4.** HRMS of pregna-5,20-dien-3β-ol (APCI^+^). p. S-12

**Figure S5.** NMR spectra of pregna-5,20-dien-3β-ol. p. S-13

**Enzymes**

**Figure S6.** SDS-Polyacrylamide gel electrophoresis of recombinant proteins p. S-14

**References** p. S-15

**Syntheses**

**Synthesis of [13-^2^H_3_]-12-oxotridecanoic acid**

**Scheme S1.** **Synthesis of [13-^2^H_3_]-12-oxotridecanoic acid.**

**Step 1. 12-Hydroxydodecanoic acid ethyl ester.** 12-Hydroxydodecanoic acid (12-hydroxylauric acid) (3.0 g, 14 mmol) was dissolved in 90 ml of absolute C_2_H_5_OH, and 2 ml of conc. H_2_SO_4_ was added. The mixture was stirred and heated under reflux overnight. Most of the C_2_H_5_OH was removed *in vacuo*, (C_2_H_5_)_2_O was added, and the (C_2_H_5_)_2_O layer (upper) was washed 3× (each) with brine, 5% (w/v) NaHCO_3_, and brine. The organic phase was dried with MgSO_4_, filtered, and concentrated *in vacuo* to yield 3.1 g of the ethyl ester (91% yield, single higher *R*_f_ spot on TLC (hexanes-ethyl acetate-CH_3_CO_2_H, 1-1-0.005, v/v/v; visualization with phosphomolybdic acid/heat).

**Step 2. 12-Oxododecanoic acid ethyl ester.** Pyridinium chlorochromate (PCC, 2.65 g, 12.3 mmol) was stirred in 20 ml of CH_2_Cl_2_, and the product from Step 1 (2.0, 8.2 mmol) was added in 5 ml of CH_2_Cl_2_. The reaction was stirred for 2 h at 23 °C, during which time the color changed from orange to black (90). The mixture was diluted with 120 ml of (C_2_H_5_)_2_O and applied to a 2 cm × 10 cm column of Florasil (packed in (C_2_H_5_)_2_O) and eluted with 80 ml more (C_2_H_5_)_2_O. The effluent was collected, dried with MgSO_4_, filtered, and concentrated *in vacuo*. TLC (hexanes-ethyl acetate, 4-1, v/v; visualization with phosphomolybdic acid/heat) showed complete reaction (to 12-oxododecanoic acid). Yield 1.6 g (85%). ^1^H-NMR (CDCl_3_) δ 9.76 (t, 1H, -CHO), 4.12 (q, 2H, -OCH_2_-), 2.42 (t, 2H, H-11), 2.34 (t, 2H, H-2), 1.27 (t, 3H, - CH_3_).

**Step 3. [13-^2^H_2_]-Tri-12-decenoic acid ethyl ester**

(a) Preparation of Wittig reagent. A 100-ml round bottom flask (with a stir bar) was flame-dried, cooled in a desiccator, and charged with 4.34 g (16.5 mmol) of triphenylphosphine in 30 ml toluene. CD_3_I (0.88 ml, 2.0 g, 14 mmol) was added and the mixture was stirred at 23 °C for 6 h. The Wittig product was collected on a filter, with vacuum, washed with 10 ml toluene and 3× with hexanes, and then dried in a vacuum desiccator. ^13^C NMR (CDCl_3_): 135.37, 133.51, 130.66 (phenyl); 119.36, 118.78 (CD_3_).

(b) A 3-neck 250-ml round bottom flask was dried in an oven (200 °C) overnight), cooled in a vacuum desiccator, swept with Ar, and charged with 100 ml of dry tetrahydrofuran and 11.7 g of the above Wittig reagent. The stirred solution (under Ar) was cooled to 0 °C, and 11.6 ml of a 2.5 M solution of *n*-butyl Li was added with a syringe. The bright red solution was stirred for 30 min at 0 °C, and then 1.6 g of 12-oxododecanoic acid ethyl ester (*vide supra*, in 10 ml tetrahydrofuran) was added dropwise. The color changed to a bright yellow, and the mixture stirred at 23 °C for 4 h (under Ar). Saturated (aq) NH_4_Cl was added to quench the reaction. Ethyl acetate was added, and the layers were transferred to a separatory funnel. The organic (upper) phase was washed 3× with brine, dried with Na_2_SO_4_, filtered, and concentrated *in vacuo*. TLC showed the disappearance of the starting material and a major high *R*_f_ spot (hexanes-ethyl acetate, 6-1, v/v, visualization with phosphomolybdic acid/heat).

The residue was dissolved in a minimal volume of CH_2_Cl_2_ and applied to a 4 cm × 17 cm silica column (SiliaFlash, 230-400 mesh). The column was eluted with 1000 ml each of 2% and 10% ethyl acetate in hexanes (v/v), and the fractions (20-25 ml each) were analyzed by TLC (hexanes-ethyl acetate, 6-1, v/v; visualization with phosphomolybdic acid/heat). The major fraction eluted with 2% ethyl acetate and was concentrated *in vacuo* to yield 1.5 g of [12-^2^H_2_]-tri-12-decenoic acid ethyl ester (39% yield), ^1^H-NMR (CDCl_3_) δ 6.80 (t, 1H, H-12), 4.12 (q, 2H, -OCH_2_), 2.28 (t, 2H, H-2), 2.04 (m, 2H, H-11). ^13^C-NMR: 173.95 (C=O), 139.04 (C-12), 61.3 (-O-CH_2_) (^13^C-13 not seen due to ^2^H substitution).

**Step 4. [13-^2^H_2_]-Tri-12-decenoic acid.** The above ester (from Step 3) was hydrolyzed by heating in 40 ml of 2.5% KOH in CH_3_OH under reflux overnight. The volume was reduced by one-half *in vacuo*, and 5% (w/v) aq. HCl was added (to lower the pH < 2). The product was extracted into ethyl acetate and the organic (upper) layer was washed 3× with brine, dried with Na_2_SO_4_, filtered, and concentrated *in vacuo*. TLC (silica, hexanes-ethyl acetate-CH_3_CO_2_H, 1-1-0.005, v/v/v; visualization with phosphomolybdic acid/heat) indicated complete hydrolysis.

**Step 5. [13-^2^H_3_,12-^2^H]-12-Hydroxytridecanoic acid** (91)**.** Mercuric acetate (2.07 g, 6.5 mmol) was stirred in a mixture of 1 ml each of H_2_O and tetrahydrofuran. The product from Step 4 ([13-^2^H_2_]-tri-12-decenoic acid, 1.5 g, 6.2 mmol) was added in 6.5 ml of tetrahydrofuran and stirred for 10 min (23 °C). During this time the original yellow color dissipated, and 2 ml of 3 M NaOH was added, followed by 2 ml of 0.5 M NaB^2^H_4_ in 3 M NaOH. The mixture (now black) was stirred for 30 min. The pH was lowered by the addition of conc. HCl (aq), and some of the mercuric salts precipitated. The mixture was filtered through glass wool and extracted 2× with ethyl acetate. The combined ethyl acetate layers were washed 3× with brine, dried with Na_2_SO_4_, filtered, and concentrated *in vacuo*. TLC (silica, hexanes-ethyl acetate-CH_3_CO_2_H, 2-1-0.005, v/v/v; visualization with phosphomolybdic acid/heat) showed the appearance of a new spot.

The crude product was dissolved in a minimum volume of CH_2_Cl_2_ and applied to a 2 cm × 15 cm silica column (SiliaFlash, 230-400 mesh), which was eluted with 250 ml portions of 4, 8, 15, and 30% ethyl acetate in hexanes (v/v, containing 0.005% CH_3_CO_2_H, v/v). TLC (*vide supra*) of the (20-25 ml) fractions indicated two major components, one eluting with 4% and 8% ethyl acetate (starting material) and the other with 15% and 30% ethyl acetate. The latter fractions were concentrated *in vacuo* and dried in a vacuum desiccator (with NaOH pellets to absorb the residual CH_3_CO_2_H). HRMS (ESI^–^) indicated that this material was [13-^2^H_2_]-12-hydroxytridecanoic acid (yield 415 mg), M-H^–^ *m/z* 232.2010 (Δ 5.3 ppm).

**Step 6. [13-^2^H_3_]-12-Oxotridecanoic acid.** [13-^2^H_3_]-12-Hydroxytridecanoic acid (*vide supra*) (189 mg, 0.81 mmol) was dissolved in 5 ml of CH_2_Cl_2_ and stirred with PCC (2.69 mg, 1.25 mmol, in 5 ml CH_2_Cl_2_) for 90 min. (C_2_H_5_)_2_O (50 ml) was added. The chromium precipitate was discarded, and the supernatant was applied to a 2 cm × 5 cm column of Florasil, which was washed with (C_2_H_5_)_2_O. The pooled fractions were concentrated and applied to a 2 cm × 12 cm silica column (SiliaFlash, 230-400 mesh), which was eluted sequentially with 200 ml portions of 4, 8, 15, and 30% ethyl acetate in hexanes (v/v, with 0.005% CH_3_CO_2_H). Analytical TLC (silica, hexanes-ethyl acetate-CH_3_CO_2_H, 4-1-0.005, v/v/v, visualization with phosphomolybdic acid/heat) indicated that the product was eluted in some of the 8% and 15% ethyl acetate fractions, which were pooled and concentrated *in vacuo* to yield [13-^2^H_3_]-12-oxotridecanoic acid. HRMS (ESI¯): M-H¯ *m/z* 230.1853 (C_13_H_20_D_3_O_3_ 230.1841, Δ 5.3 ppm), UV 192, 194 nm, broad weak absorbance at 210-230 nm. ^1^H-NMR (CDCl_3_): δ 2.42 (t, 2H, H-11), 2.35 (t, 2H, H-2). ^13^C-NMR: 209.74 (C-12 (C=O), 179.58 (C-1 (CO_2_H)), 43.96 (C-11), 34.11 (C-2).

**A**

**B**

**Figure S1. NMR spectra of 12-oxotridecanoic acid (CDCl_3_).** *A*, ^1^H-NMR; *B*, ^13^C-NMR.

**Synthesis of 11-acetoxy undecanoic acid**

11-Hydroxyundecanoic acid (100 mg) was dissolved in 3 ml of pyridine and 0.5 ml of acetic anhydride. The mixture was stirred at room temperature overnight. H_2_O and ethyl acetate were added, and the ethyl acetate (upper) layer was washed consecutively (3× each) with brine, saturated aq NaHCO_3_, brine, 5% (aq, w/v), brine, and H_2_O. The product was dried with Na_2_SO_4_, filtered, and concentrated *in vacuo*. TLC (silica, hexanes-ethyl acetate-CH_3_CO_2_H, 2-1-0.005, v-v-v) showed complete conversion to the less polar acetoxy product.

**Synthesis of 22-ooxocholesterol**

**Scheme S2. Synthesis of 22-oxocholesterol** (44,45)**.**

**Step 1.** 23,24-Bisnor-5-choleric acid-3β-ol (Fernholtz acid, 3β-hydroxy-bisnor-5-choleric acid (20*R*), 4.9 g, 14 mmol) was dissolved in 90 ml of dry pyridine and stirred on ice. Acetic anhydride (60 ml, 593 mmol) was added dropwise, with stirring, and the mixture was stirred overnight under Ar at 23 °C. The mixture was poured into ice, and the resulting mixture was extracted into ethyl acetate and the organic phase (upper) was washed sequentially, 3× with brine, 3× with 5% (w/v) HCl, 3× with brine, and 1× with H_2_O. The mixture was dried with Na_2_SO_4_, filtered, and concentrated *in vacuo* to give 4.2 g of the 3-acetyl derivative (76% yield). TLC (silica, hexanes-ethyl acetate, 2-1 (v/v), visualization with phosphomolybdic acid/heat) showed a single higher *R*_f_ spot, indicative of complete reaction).

**Step 2.** The 3-acetoxy derivative from Step 1 (1.5 g 3.9 mmol) was stirred in 60 ml of dry (C_2_H_5_)_2_O plus 50 µl of pyridine. Thionyl chloride (freshly distilled, 0.68 ml, 1.1 g, 130 mmol) was added and the reaction was stirred for 3 h at 23 ° under an Ar balloon. The solvent was removed *in vacuo*, 2 ml of benzene was added, and the solvent was removed *in vacuo* at 30 °C. Hexanes were added and the *in vacuo* drying was repeated. TLC (*vide supra*) showed a slightly higher *R*_f_ spot. The acyl chloride product was not characterized further at this stage but was dried in a vacuum desiccator and used directly in the following reaction.

**Step 3** (44). All glass was flame-dried and cooled in a desiccator. Solid magnesium turnings (Mg^0^, 0.48 g) were stirred in 36 ml of (C_2_H_5_)_2_O, with a grain of I_2_ added. 4-Bromo-1-methyl butane (isoamyl bromide, 3.6 ml, 4.3 g, 650 mmol) was added dropwise, with stirring, at 23 °C. The I_2_ color dissipated, and the solution began to reflux. After addition of the alkyl bromide, the reaction was heated under reflux for 60 min. The (Grignard) reaction was cooled on ice and CdBr_2_ (3.39 g, 12.5 mmol) was added. The mixture was stirred (0 °C) for 30 min, allowing diisoamyl Cd to form. The acid chloride from the previous step (3β-acetoxy-bisnor-5-choleric acyl chloride, *vide supra*), dissolved in 12 ml benzene, was added dropwise. The reaction was stirred for 60 min at 0 °C and then overnight at room temperature.

The stirred flask was cooled to 0 °C (ice) and then 50 ml of an ice-H_2_O mixture was added. The pH of the aqueous layer was lowered to ~ 2 with 20% H_2_SO_4_ (v/v), in H_2_O, yielding two phases. The lower phase was extracted 2× with benzene and added to the upper phase. The combined layers were washed sequentially 3× each with H_2_O, 5% NaHCO_3_, H_2_O, and brine and then dried with Na_2_SO_4_, filtered, and concentrated *in vacuo*. C_2_H_5_OH was added and removed *in vacuo*, leaving an off white solid (3β-acetoxy-22-oxocholesterol), which had a much higher *R*_f_ on TLC than the starting material.

**Step 4.** The above product (3β-acetoxy-22-oxocholesterol) was heated with a mixture of 40 ml CH_3_OH, 2 ml H_2_O, and 0.8 g Na_2_CO_3_ for 6 h under reflux. TLC showed a decreased *R*_f_ (*vide supra*). The product 22-oxocholesterol could be crystallized from CH_3_OH: yield 396 mg (three crops). mp 136-137 °C (lit 122-125 °C (45), 140-142 °C (46), 142-143 °C (44)). HRMS: MH^+^ *m/z* 401.3406 (Δ 3.5 ppm) (rel. abundance 8; MH^+^-18, *m/z* 383.3302 (Δ 1.6 ppm) (rel. abundance 100). ^1^H·NMR (CDCl_3_) δ 5.27 (t, 1H, *J* = Hz, H-6), 3.53 (m, 1H, *J* = Hz, H-3), 2.43 (m, 2H, *J* = Hz, H-23), 2.26. (m, 1H, *J* = Hz, H-20), 1.04 (s, 3H, H-21), 1.01 (s, 3H, J-19), 0.91 (s, 2H, H-25, -27), 0.84 (s, 3H, H-18). ^13^C-NMR (CDCl_3_) 215.06 (C-22), 140.72 (C-5), 121.58 (C-6), 71.75 (C-3), 56.08 (C-14), 52.05 (C-17), 50.90 (C-11).


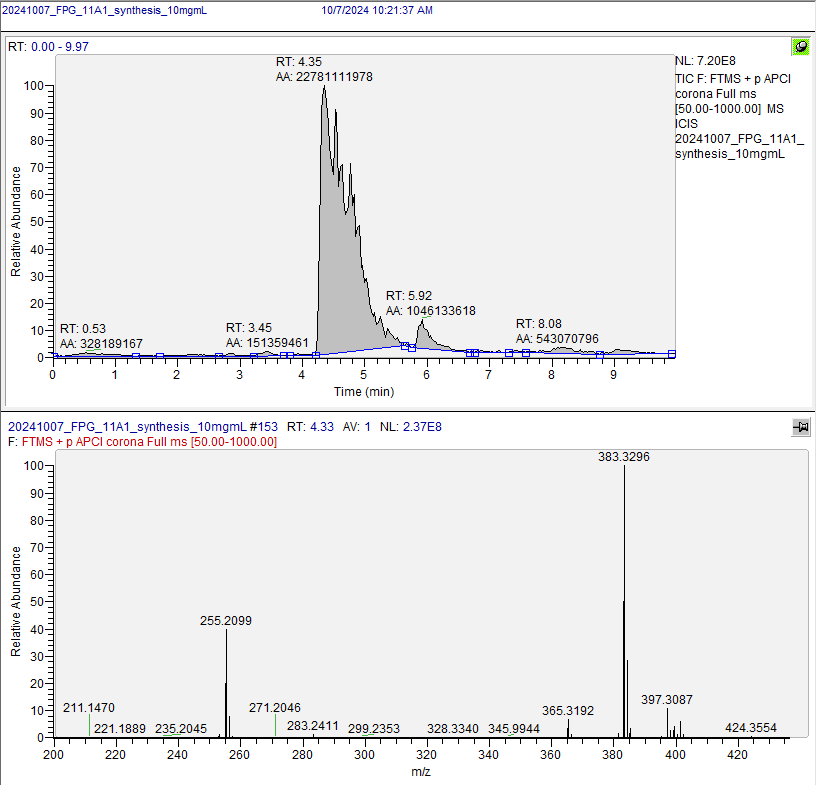


**Figure S2. HRMS of 22-oxocholesterol (APCI^+^).**

**A**

**B**

**Figure S3. NMR spectra of 22-oxocholesterol (CDCl_3_).** *A*, ^1^H; *B*, ^13^C.

**Pregna-5,20-dien-3β-ol**

**Scheme S3. Synthesis of pregna-5,20-dien-3β-ol (muricin aglycone)** (92)**.**

**Step 1.** Pregnenolone (4.74 g, 15 mmol) was dissolved in 250 ml CHCl_3_ and cooled on ice. To this was added 7.8 ml (45 mmol) of *N*,*N*-diisopropylethylamine and 0.15 g of *N*,*N*-dimethylaminopyridine (DMAP), and then 3.2 ml (3.5 g, 45 mmol) of acetyl chloride was added dropwise. The reaction was stirred for 30 min (without ice) and then heated under reflux overnight.

After cooling, the solution was washed sequentially 3× with brine (saturated aqueous NaCl), 3× with 5% (w/v) aq HCl, 2× with brine, 3× with saturated aq NaHCO_3_, and 3× with brine. The CHCl_3_ solution was dried with anhydrous Na_2_SO_4_, filtered, and concentrated *in vacuo* (reaction checked with TLC on silica (hexanes-ethyl acetate, 2-1, v/v, visualization with phosphomolybdic acid/heat)).

**Step 2.** The extract from Step 1 (3β-acetoxy pregnenolone) was dissolved in 150 ml of CH_3_OH and cooled to 0° C (ice). To this was added NaBH_4_ (570 mg, 15 mmol, 4× hydride molar excess) and the reaction was stirred at 23 °C for 1.5 h. TLC (*vide supra*) indicated that the reaction was complete. Most of the CH_3_OH was removed *in vacuo* and the residual material was dissolved in ethyl acetate. The solution was washed sequentially 2× with brine, 3× with saturated NaHCO_3_, and 3× with brine. The solution was dried with Na_2_SO_4_, filtered, and concentrated *in vacuo*. The solid could be crystallized from CH_3_OH, and the crude crystalline material (3β-acetoxy pregna-5-ene 20-ol) was used directly in the next step.

**Step 3.** 3β-Acetoxy pregna-5-ene 20-ol (2.52 g, 7 mmol) was dissolved in 46 ml CH_2_Cl_2_ and 7 ml pyridine, and mesityl chloride (CH_3_SO_2_Cl) (4.5 ml, 6.8 g, 69 mmol) was added dropwise, under an Ar atmosphere (balloon). The reaction was stirred for 18 h at 23 °C. To this was added 50 mL of saturated NaHCO_3_, and the mixture was extracted into 16 ml of CH_2_Cl_2_, which was washed sequentially 2× with saturated NaHCO_3_, brine, 5% (w/ v) HCl, and H_2_O. The solution was dried with Na_2_SO_4_, filtered, and concentrated *in vacuo*. The resulting mesylate ester was dissolved in a mixture of hexanes-ethyl acetate (1-1, v/v), applied to a 2 cm × 6 cm column of Florasil, and eluted with the same solvent mixture. The fractions containing the mesylate ester, identified by TLC (hexanes-ethyl acetate, 2-1, v/v; visualized with phosphomolybdic acid/heat), were pooled and concentrated *in vacuo*. The mesylate ester could be crystallized from CH_3_OH (yield 713 mg). LC-HRMS (APCI) showed only the ions corresponding the loss of the 3-acetate (*m/z* 379.2298, Δ1.8 ppm) and of both the 3-acetate and the mesityl group (*m/z* 283.2419, Δ 0.4 ppm, -OCOCH_3_ and -OSO_2_CH_3_) but not the parent ion (MH^+^ 439).

**Step 4.** The mesylate ester from above (3β-acetoxy pregna-5-ene 20-mesylate, 350 mg, 0.80 mmol) was dissolved in 4 ml of dry toluene and 1.9 mmol of potassium amyl alcohol (in benzene, 1.3 ml of a 1.45 M stock solution (93)) was added. The solution was heated under reflux (under Ar) for 1.5 h. The reaction was cooled and extracted into ethyl acetate, and the organic layer (upper) was washed sequentially with H_2_O and brine, and the solution was dried with Na_2_SO_4_, filtered, and concentrated *in vacuo*. The mixture was applied to a 2 cm × 16 cm silica column (230-400 mesh), which was eluted sequentially with 300 mL each of solutions of 4, 8, 15, and 30% ethyl acetate in hexanes (v/v). Screening of the eluted fractions was done with analytical TLC (silica, hexanes-ethyl acetate, 2-1 v/v, detection with phosphomolybdic acid/heat). The major product was eluted with the 8% and 15% mixtures, and these fractions were concentrated *in vacuo* to give pregne-5,20-dien-3β-ol. LC-HRMS (APCI^+^) *m/z* 283.2422 (theo. 283.2420 for MH^+^-OAc, Δ 0.71 ppm, no MH^+^ observed in APCI^+^ or ESI^+^); UV, 190 nm; ^1^H-NMR (CDCl_3_) δ 5.75 (H-20, 1H, m), 5.36 (H-6, dd, H-6), 4.96 (H22, 22´, 2H, m), 3.51 (m, 1H, H-3), 2.28 (H7, 1H, m), 1.02 (H-18, 3H, S), 0.61 (H-19, 3H, s); ^13^C-NMR: 140.8 (C5), 139.81 (C20), 121.6 (C6), 114.5 (C22), 77.1 (C3), 55.9 (C14). Although the parent ion was not seen in the mass spectrum, the NMR and UV data clearly indicate the presence of the 3-hydroxy group.


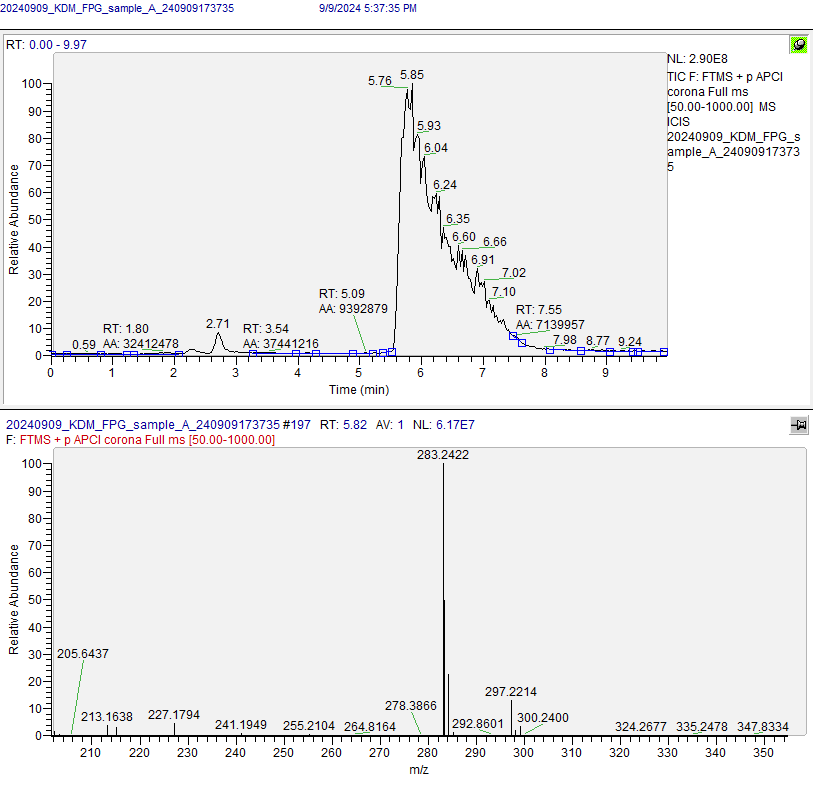
**Figure S4. HRMS of pregna-5,20-dien-3β-ol (APCI^+^).** Top, Total ion current; bottom; *t*_R_ 5.82 min spectrum.

**A**

**B**

**Figure S5. NMR spectra of pregna-5,20-dien-3β-ol (CDCl_3_).** *A*, ^1^H; *B*, ^13^C.


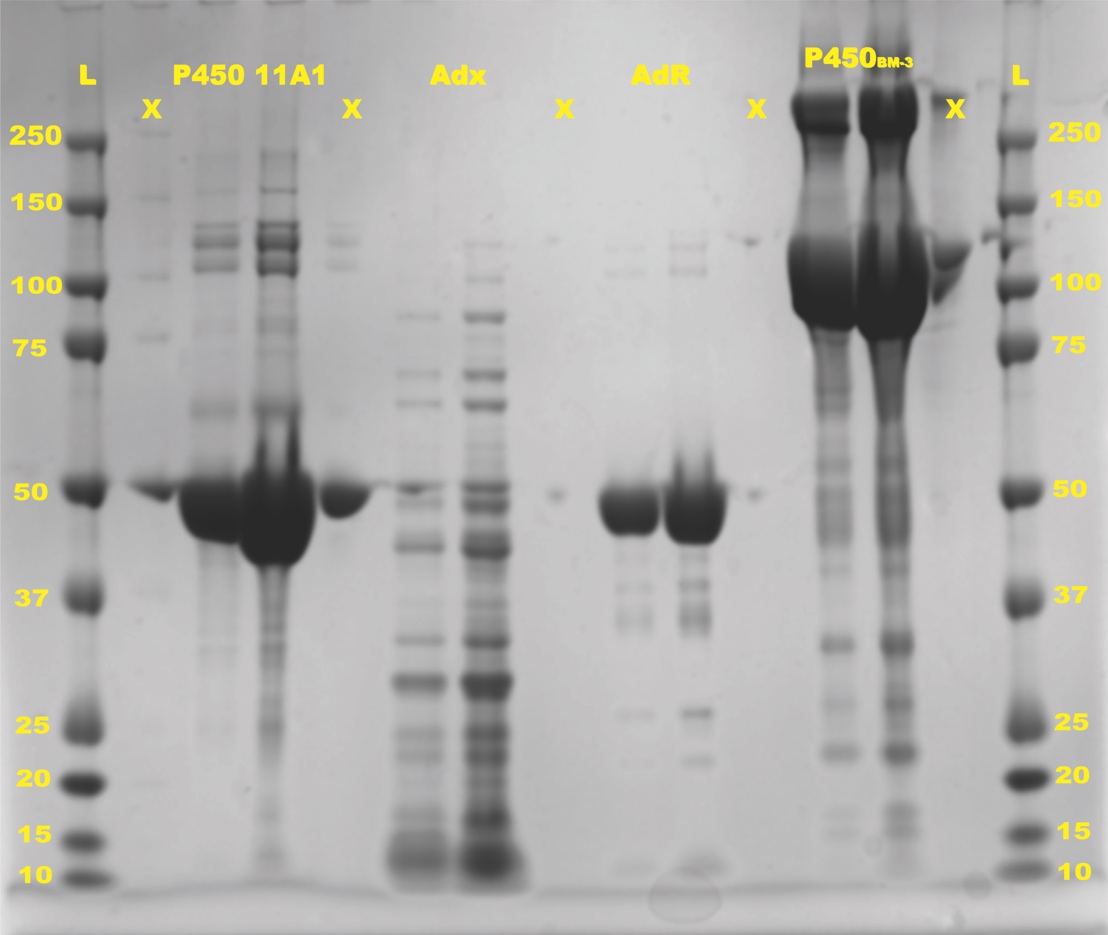


**Figure S6.** SDS-Polyacrylamide gel electrophoresis (SDS-PAGE) of recombinant proteins. A Precision Plus Protein Kaleidascope ladder was loaded on each flanking lane, and P450 11A1 (~50 kDa), adrenodoxin reductase (AdR, ~50 kDa), adrenodoxin (Adx, ~12 kDa), and P450_BM-3_ (120 kDa) were denatured in Laemmli loading dye, heated (98 °C, 10 min), and loaded (~60 and ~240 pmol amounts) on a NuPAGE 4-12% Bis-Tris polyacrylamide gel developed in K^+^ MOPS for 90 minutes at 130 V. Proteins were stained with SimplyBlue SafeStain and destained in MilliQ H_2_O. Adx was partially purified. MW ladder (L) is labeled (in kDa), protein lanes are labeled either by the protein loaded (each corresponding to two lanes) or with a “X” to indicate empty well (some lane leakage occurred).

Supporting Information References

(numbering from main text)

44. Cole, W., and Julian, P. L. (1945) Sterols. I. A study of the 22-ketosteroids. *J. Am. Chem. Soc.***67**, 1369-1375

45. Kautsky, G. J., Bouboulis, C. J., Becker, R. R., and King, C. G. (1958) Synthesis and metabolism of 22-ketocholesterol-23-C14. *J. Biol. Chem.* **233**, 1340-1342

46. Chaudhuri, N. K., Nickolson, R., Williams, J. G., and Gut, M. (1969) New synthesis of cholesterol and related C27 steroids. *J. Org. Chem.* **34**, 3767-3774

90. Corey, E. J., and Suggs, J. W. (1975) Pyridinium chlorochromate. An efficient reagent for oxidation of primary and secondary alcohols to carbonyl compounds. *Tet. Lett.***16**, 2647-2650

91. Brown, H. C., and Geoghegan, P., Jr. (1967) The oxymercuration-demercuration of representative olefins. A convenient, mild procedure for the Markovnikov hydration of the carbon-carbon double bond. *J. Am. Chem. Soc.* **89**, 1522-1524

92. Dawe, R. D., and Wright, J. L. C. (1987) An improved route to pregna-5,20-dien-3β-ol (muricin aglycone): Carbon and proton nuclear magnetic resonance assignments for the aglycone and a number of related pregnen derivatives. *Canad. J. Chem.* **65**, 666-669

93. Schow, S. R., and McMorris, T. C. (1979) Utility of the Wittig reaction for the construction of side chains of steroids starting from pregnenolone. *J. Org. Chem.* **44**, 3760-3765
